# Cardiac magnetic resonance imaging and computed tomography in ischemic cardiomyopathy: an update*

**DOI:** 10.1590/0100-3984.2014.0055

**Published:** 2016

**Authors:** Fernanda Boldrini Assunção, Diogo Costa Leandro de Oliveira, Vitor Frauches Souza, Marcelo Souto Nacif

**Affiliations:** 1 MDs, Trainees at School of Medicine - Universidade Federal Fluminense (UFF), Niterói, RJ, Brazil.; 2 MD, Resident of Magnetic Resonance Imaging and Emergency Radiology at Complexo Hospitalar de Niterói (CHN), Niterói, RJ, Brazil.; 3 Associate Professor and Vice-Chief of the Department of Radiology, School of Medicine - Universidade Federal Fluminense (UFF), Niterói, RJ, Brazil.

**Keywords:** Ischemic cardiomyopathy, Heart, Magnetic resonance imaging, Computed tomography

## Abstract

Ischemic cardiomyopathy is one of the major health problems worldwide, representing a significant part of mortality in the general population nowadays. Cardiac magnetic resonance imaging (CMRI) and cardiac computed tomography (CCT) are noninvasive imaging methods that serve as useful tools in the diagnosis of coronary artery disease and may also help in screening individuals with risk factors for developing this illness. Technological developments of CMRI and CCT have contributed to the rise of several clinical indications of these imaging methods complementarily to other investigation methods, particularly in cases where they are inconclusive. In terms of accuracy, CMRI and CCT are similar to the other imaging methods, with few absolute contraindications and minimal risks of adverse side-effects. This fact strengthens these methods as powerful and safe tools in the management of patients. The present study is aimed at describing the role played by CMRI and CCT in the diagnosis of ischemic cardiomyopathies.

## Abstract (pt)

A cardiomiopatia isquêmica é um dos principais problemas de saúde no mundo, representando significativa parcela da mortalidade. A ressonância magnética cardíaca (RMC) e a tomografia computadorizada cardíaca (TCC) são métodos de imagem não invasivos úteis no diagnóstico da doença arterial coronariana e também podem auxiliar no rastreamento de indivíduos com fatores de risco para o desenvolvimento de cardiomiopatia induzida por isquemia/infarto. Os avanços tecnológicos da RMC e da TCC contribuíram para o surgimento de diversas indicações clínicas para aplicação desses métodos de imagem de forma complementar a outros exames, principalmente quando estes se mostram inconclusivos. A RMC e a TCC apresentam acurácia semelhante aos demais métodos de imagem, poucas contraindicações absolutas e mínimos riscos de efeitos adversos, o que os fortalecem como ferramentas seguras no manejo dos pacientes. O presente estudo tem por objetivo descrever o papel da RMC e da TCC no diagnóstico das cardiomiopatias isquêmicas.

## INTRODUCTION

Ischemic cardiomyopathy is one of the major health problems worldwide, playing a significant role in the mortality nowadays. It is a condition characterized by the presence of myocardial ischemia, either associated or not with fibrosis caused by myocardial infarction. Myocardial ischemia only occurs in cases of imbalance between oxygen supply and demand, and the decreased blood flow is considered to be the physiopathology in most cases of acute myocardial infarction and unstable angina episodes. Additionally, the synergism between the oxygen supply and demand is the main determining factor of ischemia in cases of stable chronic angina^(1)^.

The clinical presentation of the coronary disease ranges from stable chronic angina to sudden death. This spectrum includes acute myocardial infarction with ST segment elevation, acute myocardial infarction without ST segment elevation, and unstable angina, currently catalogued as acute coronary syndrome^(1)^. In about 50% to 70% of patients, acute myocardial infarction is the first manifestation of ischemic cardiomyopathy^(2,3)^. The main etiopathogenic substrate of ischemic cardiomyopathies is atherosclerosis^(4)^. The risk factors for development of atherosclerosis and subsequent ischemic cardiomyopathy include, besides age, systemic arterial hypertension, diabetes mellitus, smoking, dyslipidemia, sedentarism and obesity^(5)^.

The diagnosis is based on the patient's clinical history and presence of risk factors. Electrocardiography and chest radiography may also be useful to clarify chest pain etiology. Cardiac catheterization, however, is the gold standard for the diagnosis of coronary artery disease (CAD), despite its invasiveness and expensiveness^(6)^. Currently, noninvasive cardiac imaging has been fundamental for the diagnosis and management of patients with diagnosis or even suspicious of chronic coronary disease^(7)^. Most frequently, the following noninvasive methods are utilized in the diagnosis of ischemic cardiomyopathy: exercise stress test; pharmacological stress or exercise stress echocardiography; myocardial perfusion scintigraphy; cardiac magnetic resonance imaging (CMRI); and cardiac computed tomography (CCT), with emphasis on the two latter methods^(1)^.

CMRI is extremely useful in the evaluation of CAD, both in the acute and chronic phases. Because of its high spatial resolution, this method can currently be considered as a reference standard for evaluation of the global and regional myocardial function and for detection and quantification of myocardial infarction areas^(7,8)^. CCT is a more recent method with main clinical application focused on the diagnosis of CAD, determining the Agatston coronary calcium score (CCS) and the performance of noninvasive coronary angiography^(7,8)^.

On the basis of the above considerations, the present study is aimed at describing the current concepts regarding the utilization of CMRI and CCT in ischemic cardiomyopathy, highlighting the diagnostic, therapeutic and prognostic impacts caused by the method. A systematic review was performed in the PubMed data basis sources (National Library of Medicine), utilizing the search terms "cardiac magnetic resonance in ischemic cardiomyopathy" and "computed tomography in ischemic cardiomyopathy", as well as in themecorrelated books, consensus and societies guidelines.

## RESULTS

The authors found 1319 articles and, among those, 43 articles published in high-impact journals were selected by consensus of the authors, Additionally one societies consensus and three guidelines (I Diretriz de Ressonância e Tomografia Cardiovascular da Sociedade Brasileira de Cardiologia^(8)^, III Diretriz Brasileira de Insuficiência Cardíaca Crônica^(9)^ and Diretriz de Doença Coronariana Crônica - Angina Estável^(10)^) were utilized. The present study was developed on the data collected in this review.

## DISCUSSION

### Cardiac magnetic resonance imaging (CMRI)

CMRI allows for evaluating the global and regional myocardial function, detecting and quantifying areas of myocardial infarction without utilizing ionizing radiation and nephrotoxic contrast agents, being one of the most safe methods in cardiovascular diagnosis^(8)^. Because of the obtention of ventricular volumes and masses by means of a tridimensional approach, CMRI has high accuracy in the obtention of values for both the left^(11)^ and right^(12)^ ventricles, and is considered to be the gold standard for such measurements^(7)^. Non contrast-enhanced echocardiography underestimates the ejection fraction values and volumes as compared with CMRI^(13)^. Additionally, CMRI is useful to visualize and characterize cardiac masses and thrombi, evaluate the valve function, and demonstrate with high resolution the complex processes of both congenital and acquired cardiovascular diseases^(7)^. It may also be utilized in the evaluation of the ventricular anatomy, ventricular aneurysms, pericarditis, and in the postoperative follow-up to evaluate the improvement in the cardiac function^(14,15)^.

#### Assessment of global and segmental function

**Figure 1:**
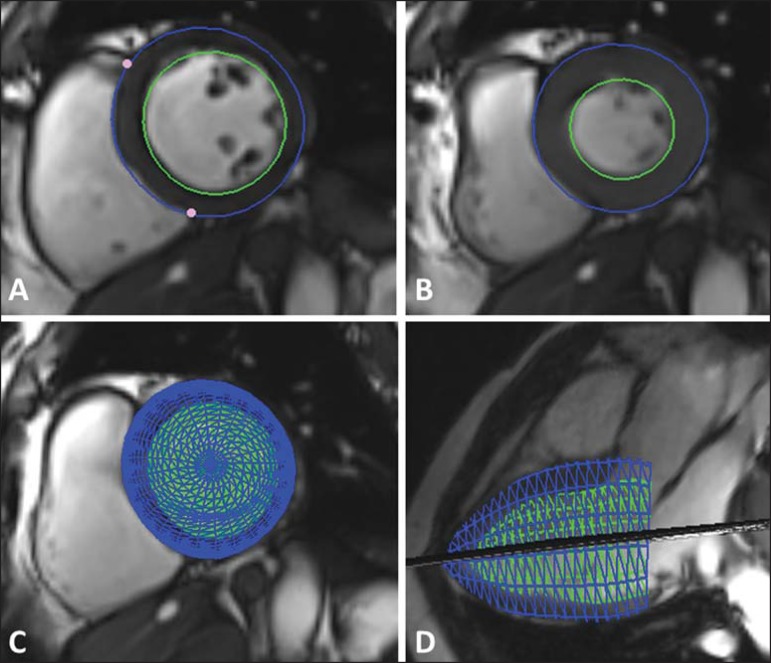
Ventricular function evaluation. Simpson's technique (**A,B**) and 4D (**C,D**).

CMRI provides accurate and highly reproducible data regarding parameters of mass, volume, and global and regional contractility of right and left ventricles^(8)^ (Figure 1). The evaluation of the regional left ventricle function (segmental contractility) is performed both at rest and under pharmacological stress. The results of the segmental contractility analysis by means of CMRI are superior to those from echocardiography^(16)^. The most utilized techniques for investigating the presence of CAD involve the direct visualization of effects from ischemia, induced by pharmacological stress, and a multimodal analysis of the segmental contractility and myocardial perfusion. CMRI presents the unique characteristic of providing both types of information in a single procedure, combining the higher specificity in the evaluation of the regional function under stress with the higher sensitivity in the assessment of the myocardial perfusion^(8)^.

#### Assessment of myocardial perfusion

**Figure 2:**
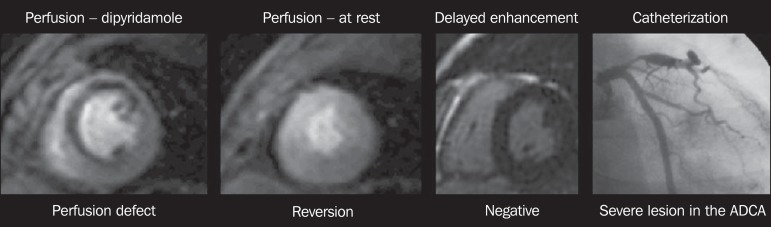
Evaluation of myocardial perfusion under pharmacological stress and at rest. ADCA, anterior descending coronary artery.

The assessment of myocardial perfusion (Figure 2) is performed at rest and under pharmacological stress (dipyridamole or adenosine) and is analyzed together with the delayed enhancement images to identify necrotic or fibrotic areas^(8)^. CMRI with myocardial perfusion has excellent sensitivity and specificity as compared with cardiac catheterization^(17,18)^. CMRI has already been validated utilizing the methods currently available in the cardiological practice with comparative analyses and longitudinal evaluation for prognostic characterization of patients. Currently, the clinical utilization of this method is already quite consolidated^(19)^. As the myocardial perfusion evaluated by CMRI under stress is normal, the patient presents with a low rate of future cardiovascular events. On the other hand, in the presence of ischemia, the rate of future cardiovascular events is high, thus determining its prognostic capacity^(20)^.

#### Evaluation of delayed myocardial enhancement

**Figure 3:**
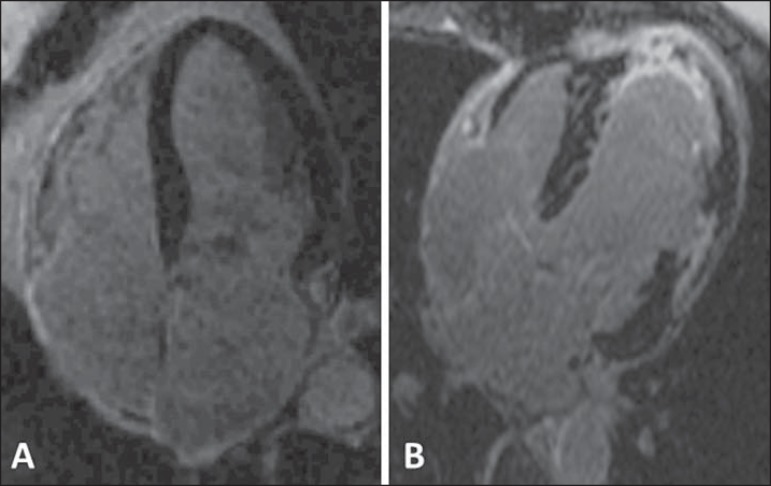
Evaluation of delayed myocardial enhancement. Normal study, black muscle (**A**). Myocardial infarction, white areas (**B**).

The protocols based on delayed myocardial gadolinium enhancement allow for accurate delimitation of the areas of myocardial necrosis or fibrosis in patients with chronic^(8)^ and acute^(7)^ infarction. The technique is based on the gadolinium property of extracellular distribution between the normal and infarcted/fibrotic tissues, with a much slower output from the latter, which generates a longer lasting contrast medium accumulation in those tissues. This allows for an evaluation of the delayed myocardial enhancement as there is a clear signal difference between the two tissues (black/ white)^(8)^ (Figure 3).

In what concerns CAD, the presence and the pattern of delayed myocardial enhancement at CMRI in a patient with ventricular dysfunction allows for the diagnosis of ischemic or non-ischemic cardiomyopathy^(7)^. The evaluation of transmurality of the regions with myocardial necrosis or fibrosis allows for predicting, with excellent accuracy, the probability of regional myocardial function recovery after revascularization, either surgical or percutaneous^(21)^. As a function of its excellent spatial resolution, CMRI can diagnose, besides transmural infarction, small subendocardial infarctions^(8)^. Cine CMRI allows for the same type of analysis of segmental contractility than echocardiography. However, as such information is combined with the detailed evaluation of the infarcted region provided by the delayed myocardial enhancement technique, CMRI allows for accurately determining what is stunned myocardial tissue and what is irreversible necrosis^(8)^.

#### Contraindications and limitations

CMRI has some relative contraindications, namely, presence of pacemakers, implanted defibrillators, cerebral clips, cochlear implants and metal fragments in the eyes^(8)^. Among the method limitations, there is the fact that coronary MRI angiography, employing several acquisition techniques, reveals moderate sensitivity (72-77%) and specificity (71-87%) values for detection of coronary stenosis as compared with invasive angiography^(22,23)^. CMRI has modest spatial resolution in the detection of coronary stenosis as compared with the high spatial resolution provided by CCT. It is possible that in the future coronary MRI becomes more usual, but currently, coronary CT angiography is a more robust method, with higher sensitivity and specificity as compared with CMRI^(23)^.

The method limitations include systemic nephrogenic fibrosis, a disease that causes systemic tissue fibrosis and is associated with the use of gadolinium in patients with chronic renal failure stages 4 and 5 and in patients with hepatorenal syndrome. It is important to highlight that such warning does not apply to patients with normal renal function. The systemic nephrogenic fibrosis physiopathogenesis is still to be understood, but it is known that there is an association between the development of the disease and use of gadolinium, which was demonstrated by the detection of gadolinium in biopsies of tissues of patients with systemic nephrogenic fibrosis, and by the disease onset after a 2-12-week period after the use of gadolinium. Systemic nephrogenic fibrosis is a quick-onset and progressive condition, determining the onset of symptoms such as muscle weakness, arthralgia, skin hardening and contractures, which, in conjunction, lead to the patient immobility. The risk for development of the disease increases at each exposure to gadolinium. For this reason, the Food and Drug Administration recommends the calculation of the creatinine clearance in the patients previously to the gadolinium enhanced CMRI, and the indication of this examination for patients at risk only if strictly necessary, followed by dialysis, despite the absence of scientific evidence that dialysis prevents the onset of systemic nephrogenic fibrosis^(24)^.

In the clinical practice, all the imaging studies for investigation of ischemia are performed with the patient under pharmacological stress, which represents a partial limitation for the non-pharmacological evaluation, in spite of the fact that some studies have already demonstrated the possibility of performing MRI with exercise stress^(25)^.

### Cardiac computed tomography (CCT)

CCT is a method that utilizes ionizing radiation and iodinated contrast agent, with the main clinical application focused on the diagnosis of CAD^(26)^. Such method presents a high negative predictive value in the detection of CAD^(9)^, and for this reason it may be utilized as an alternative to cardiac catheterization to rule out CAD^(27)^.

#### Coronary calcium score

CCT detects and quantifies coronary artery calcium, a marker of the presence and extent of atherosclerotic disease^(8)^. The presence of calcium in the coronary arteries have a strong predictive value for future cardiac events in asymptomatic patients^(28-30)^, considering the high probability of obstructive coronary disease associated with the increase in the amount of coronary calcium^(30,31)^. Thus evaluation based on the CCS allows for the differentiation between asymptomatic patients and those under risk to develop CAD over time^(7)^. At the Bethesda Conference, it was concluded that CCT and the CCS technique constitute the most accurate method currently available for early detection of coronary atherosclerosis^(7)^.

**Figure 4:**
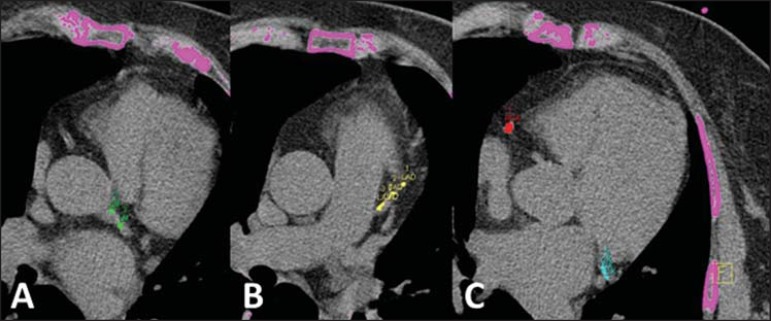
Evaluation of coronary calcium score. Left coronary trunk, green (**A**). Proximal third of the anterior descending coronary artery, yellow (**B**). Right coronary artery, red, and circumflex coronary artery, light blue (**C**).

Despite the wide applicability of the CCS, it is important to highlight that, in some situations, obstructive lesions might not contain calcium, and calcified lesions might not be obstructive^(32)^. Such process is explained by the Gagov's phenomenon^(23)^ that consists in the patency of the normal volume of the vessel, despite the presence of an atherosclerotic process, which is called positive remodelling. The evaluation of the CCS (Figure 4) is complementarily added to the clinical risk stratification data, with possibility to add clinical conducts, principally for patients considered to be at intermediate risk by the Framingham scores^(33,34)^ and by the percentile stratified by the Multi-Ethnic Study of Atherosclerosis^(35)^.

#### Coronary computed tomography angiography

**Figure 5:**
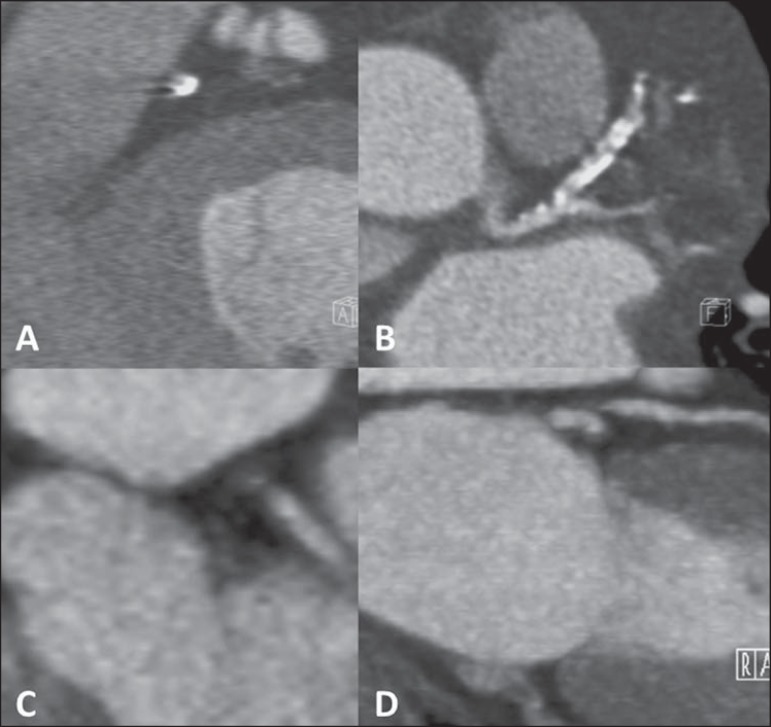
Evaluation of the vessel wall and composition of atheromatous plaques with luminal reduction by calcified plaque (**A,B**) and by noncalcified plaque (**C,D**).

**Figure 6:**
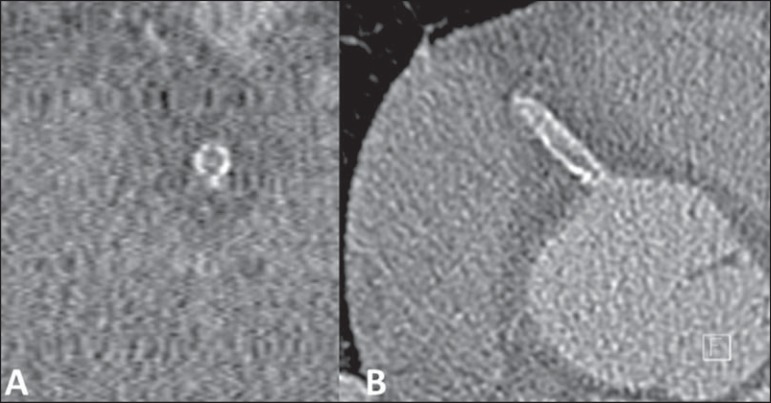
Evaluation of neointimal hyperplasia with intra-stent restenosis. Transversal axis (**A**) and longitudinal axis (**B**).

**Figure 7:**
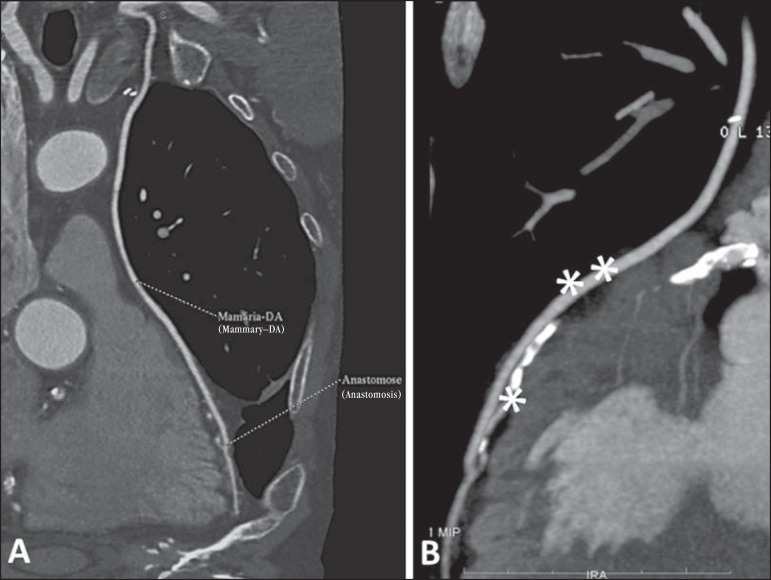
Evaluation of left internal mammary artery graft to anterior descending coronary artery (DA). Note the route of the graft in the mediastinum (**A**) and the anastomosis of the permeable graft (double asterisks) with the native anterior descending artery (single asterisk) (**B**).

**Figure 8:**
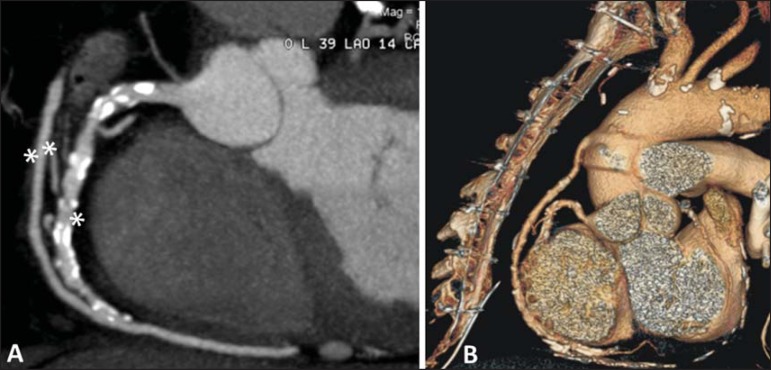
Evaluation of vena saphena graft to right coronary artery. Note the permeable graft (double asterisks) and the anastomosis with the right coronary artery (single asterisk) (**A**). 3D reconstruction (**B**).

Besides the coronary calcium evaluation, CCT may be used as a noninvasive modality of coronary angiography, which is called coronary computed tomography angiography (CCTA), with high sensitivity and specificity in the detection of coronary stenosis. It is indicated principally for patients at intermediate risk for CAD and with dubious tests for ischemia, or in patients with low clinical probability of CAD, but with positive test for ischemia^(8)^. CCTA is performed by means of multidetector CT, preferentially with 64 or more channels, under a single apnea. Routinely, the amount of contrast agent utilized is between 70 and 100 mL, which is considered to be low, therefore reducing the occurrence of problems associated with nephrotoxicity. CCTA is capable of visualizing the vessel volume and walls, which allows for a noninvasive evaluation of the presence and size of noncalcified plaques^(7)^ (Figure 5). In the evaluation of intra-stent restenosis in general, CCTA diagnostic accuracy is accepted as sufficient for clinical use in a noninvasive method, depending on the utilized method^(36,37)^ (Figure 6). The method can still be utilized to evaluate the patency of surgical grafts or for differentiation between ischemic and non-ischemic cardiomyopathy^(8)^ (Figures 7 and 8).

#### Contraindications and limitations

The main CCTA limitation occurs in the presence of dense calcification in the coronary arteries or the presence of a bare metal stent. In both situations, it will not possible to evaluate the degree of luminal obstruction. Another limitation is the necessity of a low heart rate (< 70 bpm), requiring the use of beta-blockers during the scan. The use of sublingual nitrate, as indicated, might be considered as a limiting factor as it generates a tendency to the CCTA overestimate the degree of coronary stenosis^(38,39)^, which frequently leads to the necessity for confirmation by means of a myocardial function test. The utilization of radiation is also considered to represent a limitation of the method^(7)^, but, with the recent technological developments, radiation doses have been reduced. Additionally, iodine-based contrast agents utilized in CCTA are nephrotoxic, differently from the gadoliniumbased ones utilized in CMRI. However, such a nephrotoxicity is usually self-limited and severe allergic reactions rarely occur. Preventive measures in relation to nephrotoxicity may be adopted, including a good hydration of the patient previously to the scan and use of acetylcysteine in the previous and in the following days, although studies evaluating such measures still remain controversial^(8)^.

### Future prospects

CMRI as well as CCTA are diagnostic imaging methods undergoing constant technological development.

#### Technological development of CMRI

The main future prospect for CMRI is related to the continuous improvement of the images quality, allowing for a more detailed understanding of the myocardial anatomy, function and perfusion, of the tissues characterization and cardiac viability, besides representing an important tool for clinicians and cardiologists^(40)^. Undoubtedly this will reduce the acquisition time and will maintain or improve the diagnostic potential of the method.

#### Technological development of CCT

The main prospects for CCT involve the reduction of the radiation dose affecting the patient, reduction of the acquisition time and, consequently, of the breath hold time, reduction of the volume of contrast agent required for the noninvasive acquisition of coronary arteries images, and reduction of the radiation dose. Another prospect involves the analysis of myocardial perfusion by means of dual energy CT apparatuses^(41-43)^. The high spatial and temporal resolution of this method allows for images acquisition with a great anatomical detailing and noninvasive evaluation of the spatial relation between adjacent structures^(26)^.

#### Anatomy versus ischemia/coronary flow reserve

Over the last four decades, the analysis of the coronary anatomy to predict the myocardial physiological status has ever been considered to be evidencing. However, it is currently believed that the isolated anatomical analysis cannot predict the physiological behavior of a single patient, since the biological variability is not taken into consideration. Anatomical parameters such as percentage of stenosis in the coronary diameter are not a very useful tool to understand the physiological behavior in general. In the future, the tendency is the adoption of parameters capable of more accurately and less invasively predicting the physiological status.

Such physiological parameters can be evaluated by means of noninvasive imaging methods such as PET/CT or by invasive methods such as cardiac catheterization and, recently, noninvasively by CCTA. PET/CT, because of its high spatial resolution, is capable of quantifying myocardial perfusion at rest and stress and determining the coronary flow reserve. Cardiac catheterization allows for determining the fractional flow reserve that is defined as the quotient between the pressure distal to a stenosis and the proximal pressure. Both physiological parameters allow for more accurately predicting the degree of ischemia caused by the CAD, because these parameters are superior to evaluate diffuse, multisegmental coronary diseases and those with heterogeneous remodelling. As compared with each other, the coronary flow reserve is accurate to predict ischemia and superior in the evaluation of diffuse diseases, so there is a future tendency towards the analysis of such parameter by PET/CT. The most relevant PET/CT clinical application in myocardial perfusion is the selection of patients with atherosclerosis who will benefit more from undergoing myocardial revascularization^(44)^. The calculation of the fractional flow reserve has also been studied by means of CCTA. Although still undergoing improvements, it would be theoretically ideal for the management of patients with CAD, since a single scan would allow for the anatomical evaluation of a determined stenosis and its functional, demonstrating if, in fact, there was impairment of the myocardium. However, data about clinical validation and cost-effectiveness are still limited^(45,46)^.

Another approach under study is the evaluation of myocardial perfusion under stress by CT in association with CCTA. Such a technique evaluates the myocardial perfusion um stress and the coronary anatomy, providing data about a possible ischemia and coronary stenosis, thus evaluating both the myocardial anatomy and physiology. Since 1970, there have been attempts to improve such technique, but only recently, with the technological evolution of CCT, it was possible to perform a myocardial evaluation under stress. Preliminary studies have demonstrated an improvement in the diagnostic accuracy when the techniques are utilized in combination as compared with CCTA alone. However, this technique still lacks further studies to establish imaging protocols defining contrast agents and radiation doses^(47-50)^.

## CONCLUSION

CMRI and CCT are validated as highly sensitive and specific diagnostic tools, with few contraindications and minimal risks of adverse effects, and should be utilized by physicians as aid in the management of their patients.
